# Measuring Experienced Utility in the Context of Health Economic Evaluation: A Narrative Overview

**DOI:** 10.1002/hsr2.70754

**Published:** 2025-05-21

**Authors:** Damien S. E. Broekharst, Sjaak Bloem, Jeroen Luyten, Edward A. G. Groenland, Pieter T. M. Desmet, Patrick P. T. Jeurissen, Michel van Agthoven

**Affiliations:** ^1^ Center for Marketing & Supply Chain Management Nyenrode Business University Breukelen the Netherlands; ^2^ Leuven Institute for Healthcare Policy KU Leuven Leuven Belgium; ^3^ Erasmus School of Law Erasmus University Rotterdam Rotterdam the Netherlands; ^4^ IQ Healthcare Radboud University Medical Centre Nijmegen the Netherlands; ^5^ Janssen‐Cilag B.V., Johnson & Johnson Breda the Netherlands

**Keywords:** approach‐avoidance tendencies, health economic evaluation, peak‐end perceptions, physiological indicators, retrospective impact

## Abstract

**Background and Aims:**

Expected utility is deeply ingrained in the field of health economic evaluation, but critics highlight its theoretical flaws, including assumptions of complete information, bounded rationality, and stable preferences. They propose incorporating experienced utility for greater accuracy and suggest certain measurement methods. However, the applicability of these measurement methods in health economic evaluation remains uncertain. Therefore, this article examines the advantages, disadvantages and potential use of these measurement methods in the context of health economic evaluation.

**Methods:**

The measurement methods suggested in the literature include assessing physiological indicators, peak‐end perceptions, approach‐avoidance tendencies, and retrospective impact. The advantages, disadvantages, and potential use of these measurement methods in the context of health economic evaluation are analyzed using the discourse dialectic method.

**Results:**

Evaluation of physiological indicators is minimally intrusive and relatively objective, but it relies on laboratory data collection, limits comparability across scales, and emphasizes direct experiences. Assessment of peak‐end perceptions enhances memory accuracy, yet elicits exaggerated recollections, neglects experience duration, promotes faded peak or overvalued end experiences, and disregards experiences without end. Measurement of approach‐avoidance tendencies detects implicit experiences but similarly depends on laboratory conditions, fixates on immediate automatic emotional associations, overlooks unavoidable events or states, and remains indifferent to approach‐avoidance conflicts. Evaluation of retrospective impact fosters holistic reflection and highlights temporally extended experiences, yet it fails to account for external information contamination, disregards individual rationalization processes, and overlooks constraints on reflective capabilities.

**Conclusion:**

Each proposed measurement method had drawbacks affecting its suitability for health economic evaluation. However, retrospective impact assessment emerged as the most promising one, although further scholarly inquiry is warranted to examine the theoretical and practical complexities of this approach within health economic evaluation.

## 1 Introduction

Health economic evaluation scrutinizes the intricate balance between a treatment's effectiveness and the financial outlay required for its attainment [1, 2]. Before the 1970s, the imperative of economic evaluation in healthcare was largely dismissed as ethically contentious, given its perceived commodification of human life [1, 2]. However, the escalating financial burden of healthcare in the 1980s, driven by economic turbulence, medical advancements, and increased longevity, precipitated a shift toward its broader acceptance [1, 2]. At the time, the nascent discipline of health economics lacked bespoke methods for health economic evaluation, necessitating the assimilation and refinement of prevailing evaluative methods (e.g., standard gamble, time trade‐off) from established economic realms (e.g., welfare economics and labor economics) [1, 2]. With these methods, expected utility was enshrined as a foundational principle in health economic evaluation [1, 2].

Over the last few decades, the deployment of expected utility to value treatments and determine quality‐adjusted life years (QALYs) became thoroughly ingrained in the current practice of health economic evaluation [1, 2, 3, 4, 5]. Expected utility in health economic evaluation refers to a representation of weighed preference for a given health‐related outcome on a cardinal numerical scale, where a value of 1.0 represents full health, 0.0 represents death, and negative values represent states so painful, humiliating, or otherwise burdensome that they are considered worse than death [6, 7]. Despite the tenacious and conventional insistence on the deployment of expected utility in health economic evaluation, an increasing contingent of apparent critics and detractors, especially from the fields of economic psychology and behavioral economics has gathered emphasizing the theoretical deficiencies inherent in this particular approach [2, 3, 4].

These apparent critics and detractors by the likes of Daniel Kahneman and Amos Tversky cogently remonstrate and expostulate the theoretical axioms underpinning the concept of expected utility as they are deemed questionable, ambiguous, and unrealistic [2, 8]. Firstly, they postulate that expected utility is based on an assumption of complete information, while research suggests a human inability to assemble and comprehend all available information [2, 8]. Secondly, they postulate that expected utility is based on an assumption of absolute rationality, while research shows a human proclivity to decide and act on bounded rationality [2, 8]. Thirdly, they postulate that expected utility is based on an assumption of unalterable preferences, while research indicates a human inclination to reconsider health preferences when other choice options are offered [2, 8].

To account for these theoretical deficiencies, the critics and detractors of expected utility hypothesized that health economic evaluations might become more accurate if expected utility is substituted or complemented with the measurement of experienced utility [9, 10]. Experienced utility in health economic evaluation refers to the subjective value, expressed in terms of perceived pain or pleasure, that is attributed to the cognitive, affective, and conative experience of one's own physical, mental, and social health state, which is determined through self‐reflection and interaction with significant others [2]. The concept of experienced utility is considered more theoretically sound as it relies on the direct measurement of real‐life experiences instead of the estimation of hypothetical and imprecise preferences, as is the case for expected utility [9, 10].

Although the concept of experienced utility might be theoretically robust, almost none of its proponents have proposed practical methods for its measurement. Daniel Kahneman and his colleagues stand out as exceptions, having outlined several largely theoretical measurement methods in their seminal 1997 work, *Back to Bentham? Explorations of Experienced Utility* [11]. These measurement methods, which constitute the foundation of this article, include assessing physiological indicators, peak‐end perceptions, approach‐avoidance tendencies, and retrospective impact [11]. Nevertheless, the applicability of these measurement methods within the realm of health economic evaluation remains both indeterminate and equivocal. Therefore, this article critically examines the particular advantages as well as disadvantages of these measurement methods and discusses their suitability for potential deployment in health economic evaluation.

## 2 Measurement Methods

The examination of physiological indicators, peak‐end perceptions, approach‐avoidance tendencies, and retrospective impact as preeminent measurement methods for determining experienced utility is approached through the discourse dialectic method [12]. This methodology fosters a dynamic interplay between opposing viewpoints, wherein the merits of each measurement method (thesis) are counterpoised by its limitations (antithesis), ultimately culminating in a more nuanced, refined, and logically cohesive truth (synthesis) [12].

### 2.1 Physiological Indicators

A real‐time corporal account of experienced utility could be provided by measuring physiological indicators aroused in human subjects by certain contextual stressors or events (Table 1) [11]. Physiological indicators are measurable biological functions that provide insight into the utility that is attributed by an individual to the instantaneous and direct experience of a particular event or state [13, 14]. Physiological indicators can be measured using medical examination (e.g., respiratory sinus arrhythmia, respiratory amplitude, heart rate variability) and human or technological observation (e.g., facial expression, walking speed, eye movement) [13, 14]. One might postulate that outcomes could be calibrated and valued using validated prognostic standards to generate the utility values necessary for establishing QALYs. This measurement method allows for the minimally disruptive and noninvasive determination of near impartial and objective experienced utility, reducing the risk of research burden and self‐report bias [13, 14]. However, a potential disadvantage of this measurement method is that it relies on (quasi‐)experimental conditions in laboratorial environments for data collection, which would make it rather labor‐, time‐ and resource intensive to establish a considerable data set [13, 14]. Furthermore, another potential disadvantage of this measurement method is that converting the different measurement instruments, scales, and units associated with physiological indicators (e.g., mol/m^3^, cd/m^3^, BPM) into comparable and generalizable utility values might prove to be problematic [13, 14]. Moreover, a last potential disadvantage of this measurement method is that it only assesses the experienced utility of momentary and direct outcomes without considering the experiences of temporally extended outcomes unless measurements are repeated [13, 14].

### 2.2 Peak‐End Perceptions

An accurate estimate of experienced utility could also be provided by measuring peak‐end perceptions of individuals regarding a particular event or state (Table 1) [11]. Peak‐end perceptions constitute a particular habit of memory and psychological representativeness heuristic, in which individuals tend to evaluate experiences on how they perceived its most intense point and its ending rather than based on the total sum or average of every moment of the experience [15]. Peak‐end perceptions can be measured using, for instance, VAS, ladder, rating, or other comparable scales, which allow for the recording, weighing, and adding of utility values at the peak and end of the experience of a particular event or state [16, 17]. One might argue that the average recorded utility values indicated on the aforementioned scales could be translated into QALYs. This measurement method allows for the most optimal, vivid, and evocative memorization of experienced utility employing only two focal points as well as the human proclivity for memory bias and recency bias [16, 17]. However, a potential disadvantage of this measurement method is that its focus on the peak and end of a particular experience provides a simplified snapshot of the true experience as less exciting but still important aspects of the total experience are disregarded, which often generates exaggeratedly positive or negative memories [16, 17]. Subsequently, another potential disadvantage of this measurement method is that it neglects the duration of the experience by only emphasizing the peak and end of an experience, while the duration of an experience could certainly add to or detract from its total utility [16, 17, 18]. Furthermore, a further potential disadvantage of this measurement method is that the extreme effects of peak experiences fade, and end experiences might become overvalued over time due to the shift from episodic memory into semantic memory distorting estimations of experienced utility [16, 17]. Moreover, a last potential disadvantage of this measurement method is its incorporation of end experiences, because not every state or event experienced by an individual has a clearly identifiable end, if it has one at all [16, 17].

### 2.3 Approach‐Avoidance Tendencies

A near real‐time account of experienced utility could subsequently be provided by measuring approach or avoidance tendencies exhibited by individuals towards a particular event or state (Table 1) [11]. Approach tendencies refer to a propensity to associate oneself with a desired event or state, while avoidance tendencies refer to the propensity to dissociate oneself from an undesired event or state [19]. Approach and avoidance tendencies can be measured using, for instance, approach‐avoidance tasks or implicit association tests, which record the time that it takes an individual to classify concepts or pictures into two categories [19, 20]. One might postulate that the ratios between total and reaction time might be used as utility values that could be converted into QALYs. This measurement method allows for the accurate prediction of subliminal, subconscious, and implicit experienced utility without the need for introspection, decreasing the possibility of social desirability bias [19]. However, a potential disadvantage of this measurement method is that it often makes use of simulated stimuli under (quasi‐)experimental conditions in laboratorial environments, making it difficult to implement in real‐world settings [19, 20]. Subsequently, a further potential disadvantage of this measurement method is that observed effects mainly tap into immediate and automatic emotional associations, whereas experienced utility regarding health states can arguably consist of more complex and temporally extended patterns of experiences [19, 20]. Furthermore, another potential disadvantage of this measurement method is that it assumes that certain events or states are, by definition, approachable or avoidable without considering the inexorability of certain events or states [19]. Moreover, a last potential disadvantage of this measurement method is that it does not account for approach‐avoidance conflict, which occurs when there is an event or state that has both positive and negative effects or characteristics that make the event or state appealing and unappealing simultaneously [19, 21].

### 2.4 Retrospective Impact

A rather precise and accurate ex‐post intimation of experienced utility could further be provided by measuring the retrospective impact of a particular event or state on an individual (Table 1) [11]. Retrospective impact refers to the enduring and abiding impression of a particular event or state in the past as retrospectively recalled by individuals [22]. Retrospective impact can be measured using, for instance, VAS, ladder, rating, or other comparable scales, which allow for an ex‐post assessment of the value, worth, and merit attributed to the experience of a particular event or state [22]. One might argue that the utility values indicated on the aforementioned scales could be converted into QALYs. This measurement method might allow for the comprehensive, holistic, and balanced contemplation of temporally extended experienced utility, reducing the probability of simplification bias, reality bias, and affect heuristic bias [22]. However, a potential disadvantage of this measurement method is that memories of past experiences might be distorted and contaminated by social influences, external opinions, or faulty information, which may result in, for instance, memory conformity, suggestibility bias, and confirmation bias [22, 23, 24, 25]. Furthermore, another potential disadvantage of this measurement method is that memories of past experiences might be influenced by personal interpretation, learning, rationalization, and adaptation processes, which could generate, for instance, consistency bias, hindsight bias, and rosy retrospection bias [22, 23, 24, 25]. Moreover, a last potential disadvantage of this measurement method is that it assumes individuals to have considerable reflective capabilities, while these particular cognitive abilities could certainly be limited in human subjects [22, 26].

**TABLE 1:** Measurement methods for determining experienced utility.

	Measures	Advantages	Disadvantages
Physiological indicators	Human observation; medical examination	Minimally disruptive; objective measurement	Reliance on laboratorial data collection; limited comparability of scales; emphasis on direct experiences
Peak‐end perceptions	VAS scale; ladder scale; rating scale	Memory optimalisation	Elicitation of exaggerated memories; neglect of experience duration; faded peak or overvalued end experiences; incorporation of end experiences
Approach‐avoidance tendencies	Approach‐avoidance task; implicit association test	Detection of implicit experiences	Reliance on laboratorial data collection; fixation on immediate automatic emotional associations; disregard of inexorable events or states; indifference to approach‐avoidance conflict
Retrospective impact	VAS scale; ladder scale; rating scale	Holistic contemplation; emphasis on temporally extended experiences	External information contamination; individual rationalization processes; limited reflective capabilities

## 3 Discussion

This article critically examines the particular advantages as well as disadvantages of the measurement methods for determining experienced utility proposed by Kahneman and his colleagues and ultimately discusses their suitability for potential deployment in health economic evaluation. These measurement methods include assessing physiological indicators, peak‐end perceptions, approach‐avoidance tendencies, and retrospective impact [11]. Although each of these measurement methods presents its own disadvantages, it is still debatable to what extent these disadvantages affect the suitability for deployment in the current context of health economic evaluations.

The assessment of physiological indicators has certain disadvantages that could impact its deployment in health economic evaluations, namely reliance on laboratorial data collection, limited comparability of scales, and emphasis on direct experiences [13, 14]. Research indicates that reliance on laboratorial data collection might be mitigated by providing participants with the resources to self‐assess physiological indicators in their daily life [27]. Research also shows that the limited comparability of scales could be mitigated by standardizing scales of different physiological indicators using z‐scores [28]. However, the scientific literature does not suggest a solution for the emphasis on direct experiences, making this measurement method incompatible with current practices in health economic evaluation as the effects of most health states and events are inherently extended [29, 30].

The assessment of peak‐end perceptions has multiple disadvantages that could affect its deployment in health economic evaluations, namely elicitation of exaggerated memories, neglect of experience duration, faded peak or overvalued end experiences, and incorporation of end experiences [16, 17, 18]. Research suggests that the elicitation of exaggerated memories might be mitigated by valuing the least intense point of an experience in addition to peak and end experiences [31, 32]. Research also shows that neglect of experience duration could be mitigated by providing a modulus (i.e., a standard of comparison) to participants [33]. Research further shows that faded peak experiences or overvalued end experiences could be mitigated by limiting the time between an experience and the request for recall [17]. However, the scientific literature does not suggest a solution for the incorporation of end experiences, making this measurement method incompatible with current practices in health economic evaluation as many health states or events do not have a clearly identifiable end, if they have one at all, except for death [34].

The assessment of approach‐avoidance tendencies has several disadvantages that could impact its deployment in health economic evaluations, namely reliance on laboratorial data collection, focus on immediate automatic emotional associations, disregard of inexorable events or states, and indifference to approach‐avoidance conflict [19, 20, 21]. Research suggests that the reliance on laboratorial data collection might be mitigated by using recently developed computerized and mobile approach avoidance tasks instead of the traditional laboratorial approach [35]. Research also suggests that the fixation on immediate automatic emotional associations may be attenuated by providing prompts throughout real‐life events or states to measure the inclination toward approach or avoidance over time [35]. However, the scientific literature does not suggest a solution for disregard of inexorable events or states and indifference to approach‐avoidance conflict, making this measurement method incompatible with current practices in health economic evaluation as certain health states or events are both approachable and avoidable simultaneously (e.g., pregnancy) or neither approachable nor avoidable due to their inexorable and arbitrary nature (e.g., accidents) [21].

The assessment of retrospective impact has some disadvantages that might affect its deployment in health economic evaluations, namely external information contamination, individual rationalization processes, and limited reflective capabilities [22, 23, 24, 25, 26]. Research suggests that external information contamination could be prevented to a large extent by reducing the exposure time between the experience of participants and the request to recall the memory [36]. Research also suggests that individual rationalization processes could be mitigated relatively easily by focussing the line of questioning on the primary reactions of participants to a health state or event [37]. Research further suggests that limited reflective capabilities could be prevented to a considerable degree by providing ample information and time to correctly recall the desirable memory [38, 39, 40].

In light of the preceding discussion, it can be concluded that three measurement methods for determining experienced utility, videlicet, assessing physiological indicators, peak‐end perceptions, and approach‐avoidance tendencies, have at least one or more disadvantages that might be considered irreconcilable with the theoretical tenets of and current practices in health economic evaluation. In contrast, assessing retrospective impact seems to hold the most promise, although many unresolved questions regarding its practical deployment and integration in health economic evaluation remain. Therefore, this article invites and encourages future scholarly explorations into this intriguing subject matter fostering a more profound comprehension as well as advancing academic discourse.

### 3.1 Practical Implications

The measurement and deployment of experienced utility individually or in conjunction with expected utility may hold significant relevance for health economic evaluations across a broad spectrum of diseases and associated treatments. However, certain conditions and associated treatments may be particularly well‐suited to this approach. Firstly, chronic diseases (e.g., Crohn's disease, diabetes, rheumatoid arthritis) are prime candidates as their prolonged nature places a strong emphasis on daily well‐being, quality of life, and individuals' subjective health experiences. The sustained impact of these conditions makes experienced utility a valuable metric in assessing treatment effectiveness. Secondly, mental illnesses (e.g., depression, schizophrenia, anxiety disorders) may also be a prime candidate as their severity can be distorted and amplified due to lack of familiarity, limited emotional attunement, and social stigma among the public. The profound and penetrating impact of these conditions underscores the significance of experienced utility as a vital metric in evaluating treatment effectiveness. The same holds true for other lesser‐known conditions of which the lived reality is unknown to the average individual or conditions of which the perception might be exaggerated through, for instance, fear and anxiety.

### 3.2 Future Research

This article inspires several avenues for future research that might be worth pursuing. A first avenue that might be pursued focuses on exploring different ways to derive experienced utility values from the assessment of retrospective impact, such as inferring values directly from measurement scales or indirectly from regression coefficients of health state dimensions on subjective health experience. A second avenue that may be pursued emphasizes explicating ways in which experienced utility values might be integrated with existing expected utility values, such as mapping experienced utility values onto expected utility values generated by time‐trade‐off exercises. A third avenue that could be pursued focuses on determining QALYs based on experienced utility values or a combination of experienced and expected utility values. These three avenues for future research need to be qualitatively explored using, for instance, focus groups with experts in the field as well as quantitatively tested deploying, for instance, mapping and valuation procedures.

## 4 Conclusion

Given the insights articulated in this article, it becomes apparent that each of the measurement methods for assessing experienced utility proposed by Kahneman and his colleagues has specific drawbacks that impact their applicability in health economic evaluations. Nevertheless, of the proposed measurement methods, the assessment of retrospective impact seems to hold the most promise for deployment and integration in health economic evaluation. However, further scholarly inquiry is warranted to examine the theoretical and practical complexities of this approach within health economic evaluation.

## Author Contributions

**Damien S. E. Broekharst:** conceptualization, investigation, writing – original draft, methodology, validation, visualization, formal analysis, data curation. **Sjaak Bloem:** conceptualization, funding acquisition, writing – review and editing, methodology, supervision, project administration, resources, validation. **Jeroen Luyten:** conceptualization, methodology, writing – review and editing, validation. **Edward A. G. Groenland:** conceptualization, methodology, validation, writing – review and editing, supervision. **Pieter T. M. Desmet:** conceptualization, methodology, validation, writing – review and editing. **Patrick P. T. Jeurissen:** conceptualization, methodology, validation, writing – review and editing. **Michel Agthoven:** conceptualization, methodology, validation, writing – review and editing, project administration, resources, funding acquisition.

## Conflicts of Interest

The authors declare no conflicts of interest.

## Transparency Statement

The lead author, Sjaak Bloem, affirms that this manuscript is an honest, accurate, and transparent account of the study being reported; that no important aspects of the study have been omitted; and that any discrepancies from the study as planned (and, if relevant, registered) have been explained.

## Data Availability

The authors have nothing to report.
